# Palmitoylethanolamide Mitigates Paclitaxel Toxicity in Primary Dorsal Root Ganglion Neurons

**DOI:** 10.3390/biom12121873

**Published:** 2022-12-14

**Authors:** Amira Elfarnawany, Faramarz Dehghani

**Affiliations:** 1 Department of Anatomy and Cell Biology, Medical Faculty, Martin Luther University Halle-Wittenberg, Grosse Steinstrasse 52, 06108 Halle (Saale), Germany; 2 Zoology Department, Faculty of Science, Tanta University, Tanta 31527, Egypt

**Keywords:** peripheral neuropathic pain, neurotoxicity, dorsal root ganglion neurons, palmitoylethanolamide, paclitaxel, neurite length, soma size, MTT assay

## Abstract

Chemotherapy-induced peripheral neuropathy (CIPN) is a common side effect of several chemotherapeutic agents, such as Paclitaxel. The main symptoms of CIPN are pain and numbness in the hands and feet. Paclitaxel is believed to accumulate in the dorsal root ganglia and free nerve endings. Novel therapeutic agents might help to mitigate or prevent Paclitaxel toxicity on dorsal root ganglion (DRG) neurons. Thus, we used primary DRG neurons as a model to investigate the potential neuroprotective effects of the endocannabinoid-like substance, palmitoylethanolamide (PEA). DRG neurons were isolated from cervical to sacral segments of spinal nerves of Wister rats (6–8 weeks old). After isolation and purification of neuronal cell populations, different concentrations of Paclitaxel (0.01–10 µM) or PEA (0.1–10 µM) or their combination were tested on cell viability by MTT assay at 24 h, 48, and 72 h post-treatment. Furthermore, morphometric analyses of neurite length and soma size for DRG neurons were performed. Adverse Paclitaxel effects on cell viability were apparent at 72 h post-treatment whereas Paclitaxel significantly reduced the neurite length in a concentration-dependent manner nearly at all investigated time points. However, Paclitaxel significantly increased the size of neuronal cell bodies at all time windows. These phenotypic effects were significantly reduced in neurons additionally treated with PEA, indicating the neuroprotective effect of PEA. PEA alone led to a significant increase in neuron viability regardless of PEA concentrations, apparent improvements in neurite outgrowth as well as a significant decrease in soma size of neurons at different investigated time points. Taken together, PEA showed promising protective effects against Paclitaxel-related toxicity on DRG neurons.

## 1. Introduction

Chemotherapy-induced neuropathic pain (CINP) is a dose-limiting side effect of some anticancer drugs, such as bortezomib, cisplatin, oxaliplatin, paclitaxel, thalidomide, and vincristine [1]. The incidence of CINP in patients ranges from 12.1% to 96.2%, depending on the chemotherapeutic agent used and the type of cancer treated [2]. Taxanes are a class of chemotherapy drugs that promote tubulin polymerization into highly stable intracellular microtubules and cause cell death by intermixing with microtubules via normal cell division [3,4]. Paclitaxel is a Taxane derivative that has been used successfully as a first-line treatment for a variety of solid tumors, including ovarian cancer, breast cancer, cervical cancer, lung carcinomas, and other solid tumors [5,6,7].

Unfortunately, peripheral neuropathic pain (PNP) is a common side effect of Paclitaxel treatment affecting around 70.8% (95% CI 43.5–98.1) of patients [8]. The incidence ranges from 30 to 50% after a single dose and rises to more than 50% after a second dose [9]. Hyperalgesia, allodynia, and sporadic burning, shooting, numbness, spasm, and prickling sensations are some of CINP signs, and these can drastically lower the patient’s quality of life [10,11]. Chemotherapy-induced peripheral neuropathy (CIPN) is predominately a sensory axonopathy and neuronopathy, and the sensory neurons residing in dorsal root ganglions (DRGs) are the primary targets. Therefore, DRG explants have been shown to represent a good, simple, and well-accepted model for studying peripheral neuropathy induced by antineoplastic agents [12]. The ability of DRG explants to outgrow neurites in vitro when exposed to nerve growth factor (NGF), as well as the interference with neurite elongation by toxic substances, is the basis for their use in drug neurotoxicity assessment [12,13,14].

The neurotoxic effect of Paclitaxel on neurite length of DRG was shown to be dose- and time-dependent [15,16], and DRG dissociated post-mitotic neurons were observed to die by necrosis [15]. Paclitaxel also caused the enlargement of neuronal cell bodies, and suppression of DRGs neuritis [17]. Paclitaxel has shown to demonstrate concentration- and time-dependent effects on vesicular trafficking and membrane localization of Nav1.7 in sensory axons of DRGs, providing a possible mechanistic explanation for increased excitability of primary afferents and pain [18]. Paclitaxel was reported to alter intracellular trafficking in both *Drosophila* and mouse models of CIPN by inducing recycling defects in mouse DRG neurons in vitro [19]. Currently, tricyclic antidepressants and analgesic drugs such as amitriptyline, morphine, gabapentin, and duloxetine display limited efficacy for preventing and alleviating paclitaxel-induced peripheral neuropathic pain and/or suffering of patients from serious side effects [20,21,22,23]. As a result, finding novel therapeutic agents that can mitigate or prevent Paclitaxel neurotoxicity on DRG neurons is very crucial.

The endocannabinoid system (ECS) is an important biological system that regulates and balances a wide range of physiological functions in the body, making it a target for many drugs and therapies [24]. Modulating the ECS activity showed promising therapeutic effects in a wide range of diseases and pathological conditions, including neurodegenerative, cardiovascular, and inflammatory disorders, obesity/metabolic syndrome, cachexia, chemotherapy-induced nausea and vomiting, tissue injury, and pain [25]. Palmitoylethanolamide (PEA), an endogenous fatty acid amide analogue of the endocannabinoid anandamide, has an important role in tissue protective mechanisms [26,27]. PEA was discovered nearly 5 decades ago in lipid extracts of various natural products, and its anti-inflammatory and antinociceptive properties were later described [28].

There is evidence for PEA synthesis during inflammation and tissue damage. PEA has a variety of beneficial effects, including the relief of inflammation and pruritus, and is effective in the control of neurogenic and neuropathic pain [29]. The hypothesized theories for PEA’s mode of action include modulating endocannabinoid signaling and indirectly activating cannabinoid receptors via “entourage” effects [30,31,32,33].

PEA acts primarily through the direct activation of the nuclear receptor PPAR-α [34]. After the activation of PPAR-α receptor, a chain of events leads to suppression of pain and inflammatory signals, including the inhibition of the release of pro-inflammatory cytokines such as IL-1β and IL-6 [35]. Previous studies showed a PEA-mediated protection of dentate gyrus granule cells during secondary neuronal damage, which was mediated by PPAR-α activation and influenced by reduction in inflammatory processes [36]. In a chronic constriction injury model of neuropathic pain, repeated PEA treatment (30 mg/kg) not only decreased edema and macrophage infiltrates, but also declined the decrease in axon diameter and myelin thickness [37]. However, research on studying the protective role of PEA against the toxicity of Paclitaxel on DRG neurons is still lacking.

In the present study, the effects of different Paclitaxel and PEA concentrations were investigated, either individually or in combination, on the viability, morphology, and neurite length of primary DRG neurons at various time points. We hypothesized that PEA might reduce the neurotoxicity induced by Paclitaxel on DRG neurons in a concentration- or/and time-dependent manner.

## 2. Materials and Methods

### 2.1. Ethics Statement

All animal experiments were carried out in accordance with the policy on ethics and the policy on the use of animals in neuroscience research, as specified in directive 2010/63/EU of the European Parliament and of the Council of the European Union on the protection of animals used for scientific purposes and were approved by local authorities for laboratory animal care and use (State of Saxony-Anhalt, Germany, permission number: I11M27).

### 2.2. Materials

Experiments were conducted with Palmitoylethanolamide (PEA, Tocris Bioscience, cat No. 0879-10 mg, Bristol, UK), Paclitaxel (Taxol equivalent, Invitrogen, cat No. P3456-5 mg, Schwerte, Germany), Nerve Growth Factor-2.5S from the murine submaxillary gland (NGF, Sigma Aldrich, Merck, cat No. N6009-4X 25 µg, St. Louis, MO, USA) and glial cell-derived neurotrophic factor (GDNF, Sigma-Aldrich, cat No. SRP3309-10 µg, St. Louis, MO, USA), Uridine (Uridin, Sigma-Aldrich, U3003-5 g, Darmstadt, Germany), and 5-Fluoro-2-deoxyuridine (FudR, Sigma-Aldrich, cat No. F0503-100 mg, Darmstadt, Germany). PEA and Paclitaxelwere dissolved in DMSO to obtain stock solutions of 10 mM PEA and 1 mM Paclitaxel and stored at −20 °C, while NGF and GDNF dissolved in 0.1 % Bovine Serum Albumin (BSA, Sigma-Aldrich, cat No. A7906-10 g, St. Louis, MO, USA). A total of 20 mM uridine/5-fluorodeoxyuridine (UFdU) stock solution was prepared by mixing 48.8 mg uridine and 49.2 mg 5-fluorodeoxyuridine in 10 mL distilled water, and 100 μL aliquots were prepared and frozen at −20 °C. Notably, controls contained the similar highest concentration of DMSO (0.1%) to exclude any effects on investigated parameters.

### 2.3. Isolation and Preparation of DRG Neurons

DRG tissues isolated from 6–8 weeks of age Wister rats. In brief, rats were deeply anesthetized with isoflurane (Florene, 100% (*V*/*V*), 250 mL, Abcam, cat No. B506, Carros, France) by inhalation and sacrificed by decapitation with a commercial guillotine. Under aseptic conditions, the vertebral column was isolated and carefully cleared from all surrounding muscle, fat, and other soft tissue. The spinal cord was then exposed and scooped out. Following the dorsal roots. DRGs were localized, removed, collected from intervertebral foramina at both sides, and placed in a 3 mL sterile dish containing Hanks balanced salt solutions without Mg^2+^/Ca^2+^ (HBSS, Invitrogen, REF. 24020-091, Schwerte, Germany). Dorsal root neuronal culture was prepared according to a previously published protocol [38] with some modifications. Briefly, isolated DRGs were enzymatically digested in the first enzymatic solution, which contained 60 U/mL papain solution (Sigma-Aldrich, cat No. P4762-100 mg, St. Louis, MO, USA), 3 µL of 80 mg/mL saturated sodium hydrogen carbonate solution (NaHCO3, Merck, cat No. k22399729, Darmstadt, Germany), and 0.6 mg/mL L-Cysteine (L-Cys, Sigma-Aldrich, Cat No. C7352-25 g, St. Louis, MO, USA) dissolved in 1.5 mL of HBSS without Mg^2+^/ Ca^2+^. Afterwards, DRGs were incubated in a papain solution for 15 min in a 37 °C water bath, then incubated in a second solution which consisted of 4 mg/mL collagenase type II solution (CLS2, Sigma-Aldrich, Cat No.C6885-1 gm, St. Louis, MO, USA) and 4.6 mg/mL dispase type II (Dispase II, Sigma-Aldrich, Cat No. D4693-1 gm, St. Louis, MO, USA) solution in 3 mL HBSS without Mg^2+^/ Ca^2+^. The DRGs were mixed gently in collagenase solution and incubated again for 15 min in a water bath at 37 °C.

The resulting cell suspension was centrifuged at 200× *g* for 1 min. The collagenase solution was carefully aspirated, and the DRGs were washed with 2ml of titration media consisting of high glucose Dulbecco’s Modified Eagle Medium (DMEM, Invitrogen, Ref. 41965-039, Schwerte, Germany) containing 10% heat-inactivated Fetal Bovine Serum (FBS, Invitrogen, REF. 10270-106, Schwerte, Germany). The DRGs were triturated 10–15 times by using p1000 pipette tips until the cell suspension became cloudy. Bovine serum albumin (BSA) was used for purification (15% (*W*/*V*) BSA solution) to obtain nearly pure neurons without myelin debris. After trituration, single-cell suspensions from DRGs were centrifuged through 15% (*W*/*V*) BSA solution in DMEM, 3 mL of 15% BSA solution: 1 mL of cell suspension in a 15 mL conical tube at 300 g for 8 min at room temperature (RT) to separate sensory neurons in the pellet from non-neuronal cells and debris [39]. The BSA solution was removed and the pellet containing neurons was re-suspended in 1 mL of culture medium consisting of 445 mL of F12 medium (1X, Invitrogen, REF.11765-054, Schwerte, Germany), 50 mL of FBS and 5 mL of 0.1 mg/mL streptomycin/penicillin (Sigma Aldrich, cat No. P4333/100 mL, Darmstadt, Germany). The cell suspension was filtered by a 40 µm cell strainer (SARSTEDT, cat No. D-51588, Schwerte, Germany) to obtain single-cell suspensions and remove undigested tissue debris.

### 2.4. Seeding and Growth of DRG Neurons

**Figure 1:**
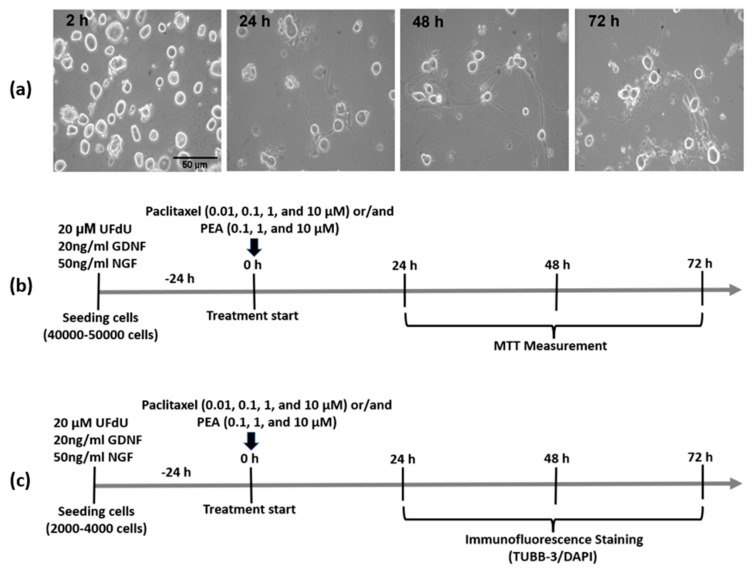
Morphological features and treatment protocols of DRG neurons. (**a**) Representative images show the morphology and growth of DRG neurons at different time points after BSA purification. Scale bars = 50 µm. (**b**,**c**) treatment protocols for studying the effects of Paclitaxel or /and palmitoylethanolamide (PEA) on DRG neurons viability and morphology (Neurite length measurement) respectively, at 24, 48, and 72-h post-treatment.

Coverslips 12 mm round were pre-coated with 2 mg/mL Poly-D-lysine (PDL, Sigma Aldrich, cat No. P6407, St. Louis, MO, USA) and 0.2 mg/mL laminin (Sigma Aldrich, cat No. L2020-1 mg, St. Louis, MO, USA) for at least 1 h or overnight in 4 °C, then washed one time with distilled H_2_O directly before seeding the cells in culture medium. DRG neuronal cells (5000 cells in 50 µL culture medium) were then pre-seeded onto the center of the coated coverslips for 2 h in an incubator with 37 °C and 5% CO_2_. Then, 1 mL of warm culture medium adjusted at pH 7.4 containing 50 ng/mL NGF and 20 ng/mL GDNF (which is essential for growing neuritis of neurons) and 20 µM UFdU (for inhibiting the growth of any remains of supporting cells in culture) was gently added to the wells, and the cells were maintained again at 37 °C with 5% CO_2_. The growth and morphology of neurons were monitored after 2, 24, 48, and 72 h to detect the suitable time of treatment (Figure 1a).

### 2.5. Cell Viability (MTT Assay)

DRG neurons were treated 24 h after seeding. Cells were treated with different concentrations of Paclitaxel (0.01, 0.1, 1, 10 µM) and PEA (0.1, 1, 10 µM), either individually or simultaneously combined to study the effects on cell viability. Paclitaxel concentrations were selected based on the literature [15,16,17,18,19,40,41] as well as PEA [42,43,44]. DRG neurons (4–5 × 10^4^ cells/well) in 96 well plates were treated with different concentrations of Paclitaxel and PEA alone or in combination for 24, 48, and 72 h. (Figure 1b). Then, cell viability (%) was measured at the different time points using MTT assay. Four hours before termination of experiments at different time points, 3-(4,5-dimethylthiazol-2-yl)-2,5-diphenyltetrazolium bromide solution (MTT, Invitrogen, cat. No M6494, 5 mg/mL, Eugene, OR, USA) was added. Cells were further incubated for 4 h at 37 °C and 5% CO2. After removing MTT solution, formazan crystals dissolved in 100 µL of dimethyl sulfoxide (DMSO, Sigma-Aldrich, cat No. D4540-500 mL, Lyon, France) were added and, after another 20 min absorbance values, were measured at wavelengths (540 nm and 720 nm) by a microplate reader (SynergyTMMx, BioTek Instruments, Winooski, VT, USA). DRG neurons cultured in normal media free of Paclitaxel or/and PEA were used as control groups. Controls contained the similar highest concentration of DMSO (0.1%) to exclude any solvent effects on cell viability. All experiments were performed three times independently with 2–3 technical replica for each treatment.

### 2.6. Immunofluorescence Staining and Microscopy

To investigate the effects of various treatments on the morphology of DRG neurons, cells (2–4 × 10^3^ cells/well) were seeded on 12 mm sterile coverslips in a 24-well plate (Greiner Bio-One, Cat No. 662160, Frickenhausen, Germany), cultured for 24 h until most neurites outgrew, and then treated with different concentrations of Paclitaxel or PEA, either alone or in combination (Figure 1c). At the end of each time point, the cells were fixed with 4% paraformaldehyde (PFA, AppliChem, cat No.141451.1211, Darmstadt, Germany) for 15 min at RT and immediately subjected to immunofluorescence or stored in 1 × PBS at 4 °C until further use. For immunofluorescence staining, fixed cells were washed 3 times with 0.02 M PBS for 10 min before unspecific bindings were blocked by incubating cells in normal goat serum (NGS, Sigma Aldrich, cat No. G9023-10 mL, Taufkirchen, Germany, 1:20) in 0.02 M PBS/0.3% (*v*/*v*) Triton) for 30 min. Afterward, cells were incubated with neuronal marker mouse anti-β-III tubulin antibody (TUBB3, Biolegend, San Diego, cat No: 801201, CA, USA, 1:1000) overnight for labelling the cytoskeleton of neurons. Coverslips were thereafter washed thrice for 10 min in PBS, incubated with the secondary antibody goat anti-mouse Alexa Fluor^®^ 488 conjugated (Life Technologies, cat No. 2066710, Darmstadt, Germany, 1:200) for 1 h, washed again 3 times with PBS, and stained with DAPI (4′,6-Diamin-2-phenylindol, Sigma-Aldrich, Munich, Germany, cat No. D9542) for visualization of nuclei. The stained cells were washed in distilled water and covered with DAKO fluorescence mounting medium (DAKO, Agilent Technologies, Inc., Santa Clara, CA 95051, USA). The DRG neurons photomicrographs were captured by using a Leica DMi8 (Wetzlar, Germany) microscope, and five images were randomly taken from each coverslip. The experiment was performed 3 times independently.

### 2.7. Image Analysis and Determination of Neurite Lengths and Soma Sizes

Measurement of neurite length as a marker for investigating the neurotoxicity of DRG neurons was assessed by using Neurite Tracer, a plugin for ImageJ software version v1.52 used for automated neurite tracing as previously described [45] with some adjustments (Figure S1). Briefly, a sample image pair from cultures of DRG neurons fluorescently labelled with TUBB3 as neuronal marker and DAPI as nuclear marker were opened in Image J (v1.46r (National Institutes of Health, Laboratory for Optical and Computational Instrumentation, University of Wisconsin, Madison, WI, USA) and converted to 8-bit grayscale, and then individually opened in neurite tracer plugin. Large bright objects (somats of neurons) were removed from all images by application of Fiji software version 2.9.0 (accessed 15 January 2022) (https://imagej.net/Fiji/Downloads). Thereafter, the resulting images were inserted to neurite tracer. Afterwards, the threshold was adjusted manually before starting the automated tracing of neuritis. Images with the traced neuritis were merged with RGB original images to ensure the reliability and accuracy of the tracing process. Afterwards, the number of neurons was determined by using a multi-point tool of ImageJ. Finally, traced neuritis lengths were normalized with the numbers of neurons to calculate the neurite length/cell. To determine the size of neuronal somata, soma areas of neurons were selected, and soma areas were measured. The results were normalized with those from the control group.

### 2.8. Statistical Analysis

Data analysis and visualization were carried out by using GraphPad Prism (GraphPad Software version 8.0.1 for Windows, La Jolla, CA, USA). The normal distribution of data was assessed by use of the Kolmogorov–Smirnov test. The effect of treatments on viability and neurite length of DRG neurons was assessed using one-way ANOVA (analysis of variance) followed by the Bonferroni multiple comparisons test (*p* < 0.05). An alpha level of 0.05 was used for all tests.

## 3. Results

### 3.1. Characterization of DRG Neuronal Cells

DRG neurons cultures were examined under a light microscope at various time points (2, 24, 48, and 72 h) to track their growth and morphology. After 2 hours, neuron somas appeared round, bright, and refractile, with a large nucleus (Figure 1a). Three distinct subpopulations (small, medium, and large neurons) based on soma diameter were observed, (Figure 1a). Most of the DRG neurons extended long thin neuritis after 24 h of cells seeding, while, after 48 and 72 h of culturing, all sensory neurons had long neuritis which connected and formed networks together (Figure 1a).

### 3.2. Effects of Paclitaxel or/and PEA on Cell Viability of DRG Neurons

**Figure 2:**
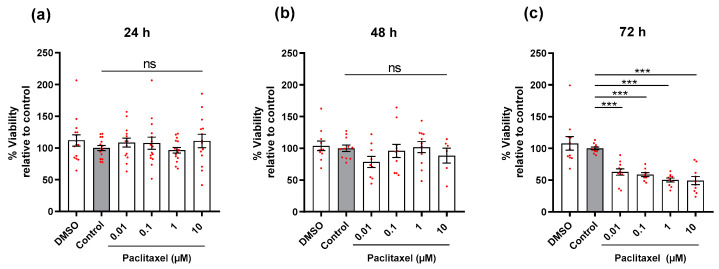
Effects of different concentrations of Paclitaxel on viability (%) of DRG neurons at different time points. Application of different concentrations of Paclitaxel showed no influence on the viability of neurons at (**a**) 24 h and (**b**) 48 h, whereas, at (**c**) 72 h post-treatment, Paclitaxel significantly reduced the viability of cells compared to control (*** *p* < 0.001). The asterisk denotes significant results regarding the respective measurement indicated with the bar. Values are served as mean ± SEM of three independent experiments performed in triplicate. SEM: Standard error mean.

DRG neurons were treated with different concentrations of Paclitaxel for 24, 48, and 72-h, and we found a significant reduction in the viability of cells at only 72 h post-treatment, regardless of Paclitaxel concentrations, compared to the untreated control group (*p* < 0.001) (Figure 2). Paclitaxel‘s effects on neuron viability were obviously time-dependent but not concentration-dependent. PEA, as expected, showed no statistically significant effect on the viability of cells in comparison to the untreated control group (*p* ˃ 0.05) at 72 h post-treatment (Figure S2).

**Figure 3:**
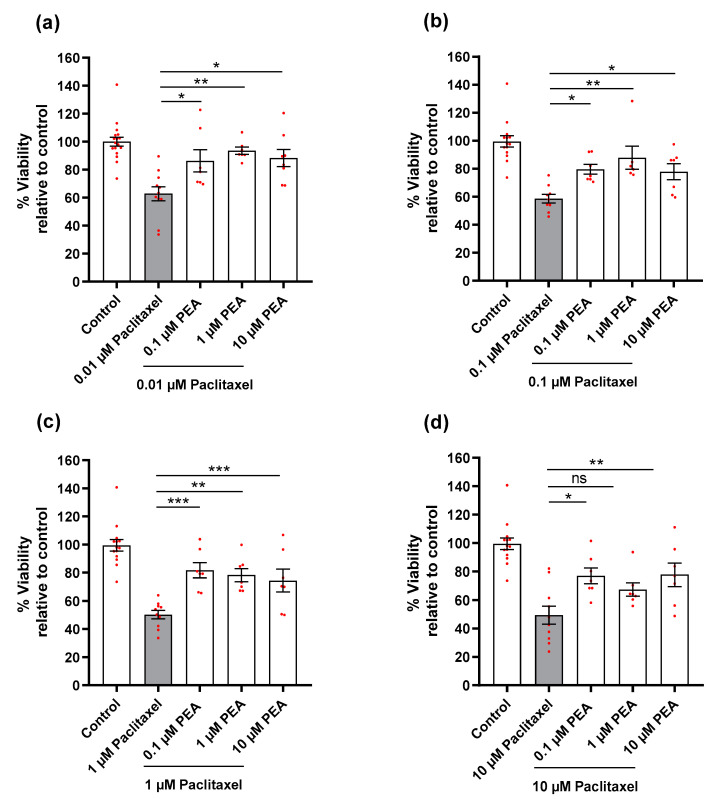
Effects of different concentrations of Paclitaxel (**a**) 0.01 µM, (**b**) 0.1 µM, (**c**) 1 µM, and (**d**) 10 µM either alone or in combination with different concentrations of PEA on viability (%) (mean ± SEM) of DRG neurons at 72 h post-treatment by using MTT assay. The asterisk indicates a significant increase in viability of DRG neurons treated with different Paclitaxel concentrations in combination with different concentrations of PEA at 72 h post-treatment compared to cells treated with Paclitaxel only (* *p* < 0.05, ** *p* < 0.01, and *** *p* < 0.001). Data are (mean ± SEM) of three independent experiments performed in duplicate.

The effects of combined treatments (Paclitaxel plus PEA) were compared to the effect of Paclitaxel alone on viability (%) at 72 h post-treatment. A significant increase was observed for almost all combinations of Paclitaxel (0.01–10 µM) plus PEA (0.1–10 µM) compared to cells treated with Paclitaxel alone. A significant effect was missed only for the combination (10 µM Paclitaxel + 1 µM PEA vs. 10 µM Paclitaxel) (*p* < 0.05) (Figure 3). Notably, the effect of PEA against Paclitaxel was clearly concentration independent.

### 3.3. Effects of Paclitaxel or/and PEA on Morphology of DRG Neurons

**Figure 4:**
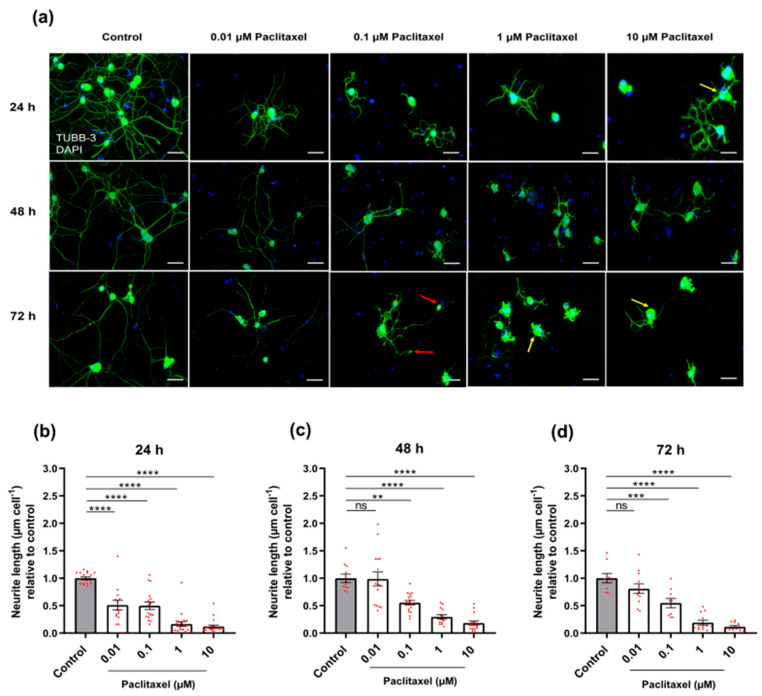
Effects of different Paclitaxel concentrations on morphology, neurite length, and soma size of DRG neurons. (**a**) Immunofluorescence staining of DRG neurons treated with different Paclitaxel concentrations (0.01, 0.1, 1, and 10 µM) labeled with anti-mouse beta III Tubulin antibody after 24, 48, and 72 h. Different concentrations of Paclitaxel had toxic effects leading to a reduction in neurite length and an increase in soma area (yellow arrows) at all time points. Additionally, other characteristics of Paclitaxel toxicity on neuronal morphology are visible, including swellings and blebbing at distal ends of neuritis (red arrows). Nuclei were counterstained with DAPI. Five to eight regions were recorded randomly per each coverslip. Scale bars = 75 µm. (**b**–**d**) significant suppression in neurite lengths of DRG neurons treated with different Paclitaxel concentrations in comparison with the control group (** *p* < 0.01, *** *p* < 0.001, **** *p* < 0.0001) at 24, 48, and 72-h post-treatment, respectively. Data are (mean ± SEM) of three independent experiments performed with (10–15) replicates.

Toxic hallmarks of Paclitaxel were observed on the morphology of neurons such as suppression in neurite lengths of neurons, swelling of neuronal cell bodies, as well as retraction and blebbing formation at the distal endings of neurites (Figure 4a). To verify and quantify the Paclitaxel and PEA effects, two different endpoints were assessed, namely neurite length and soma size.

#### 3.3.1. Neurite Length

The treatment with the four different concentrations of Paclitaxel resulted in a significant reduction in neurite length 24 h after treatment when compared to the non-treated control group (*p* < 0.05) (Figure 4b). At 48 and 72 h post-treatment, all studied Paclitaxel groups had an apparent reduction in neurite length except for 0.01 µM Paclitaxel relative to control group (Figure 4c,d). Interestingly, Paclitaxel effects on neurite length were clearly time- and concentration-dependent. No alterations in morphology and neurite length were found in DRG neurons treated with PEA in comparison to the vehicle control group (*p* > 0.05) (Figure S3a–c).

The three different concentrations of PEA co-applied with 0.01 µM Paclitaxel had no significant protective effects on the neurite lengths of neurons at all investigated timelines compared to the 0.01 µM Paclitaxel group (*p* > 0.05; Figure S4). All combined groups of PEA with 0.1 µM Paclitaxel did not cause any significant increase in neurite length at any time point when compared to the 0.1 µM Paclitaxel group alone (*p* > 0.05); however, at 72 h after application, 1 µM PEA only plus 0.1 µM Paclitaxel resulted in an apparent increase in neurite length of DRG neurons compared to 0.1 µM Paclitaxel group (*p* < 0.05; Figure S5).

**Figure 5:**
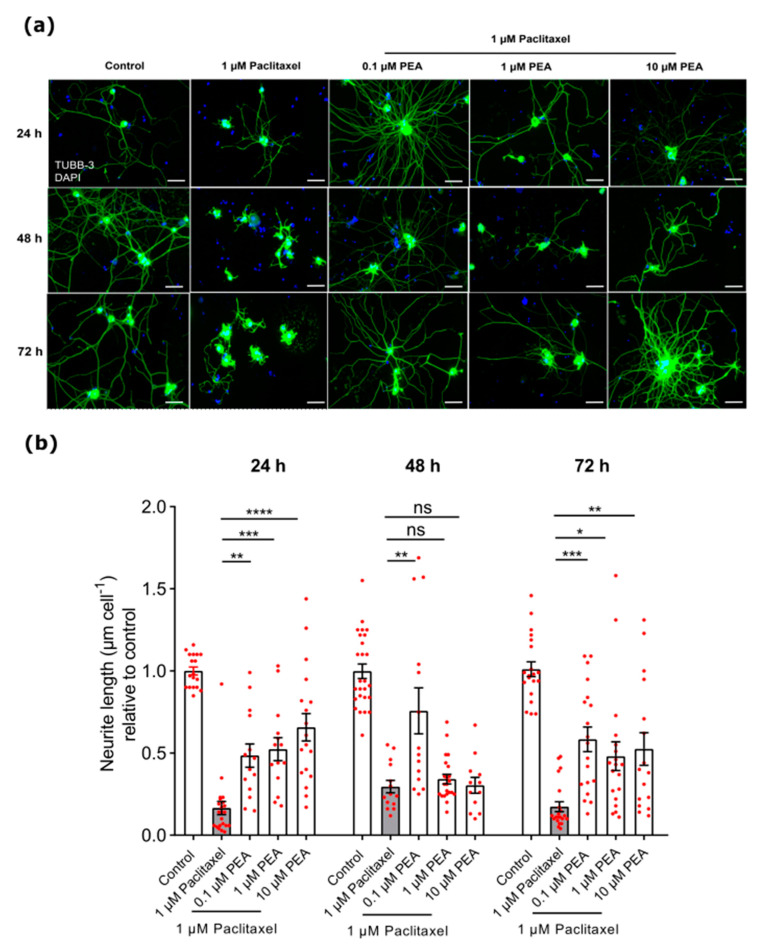
Showing protective effects of different PEA concentrations (0.1, 1, and 10 µM) co-applied with 1 µM Paclitaxel on neurite lengths of DRG neurons compared to 1 µM Paclitaxel alone at 24, 48, and 72 h post-treatment. (**a**) Representative microphotographs of DRG neurons stained with beta III Tubulin antibody for soma and neuritis (green) and DAPI for nuclei (blue). Scale bars = 75 µm. (**b**) Bar graphs indicated a significant increase in neurite lengths of neurons treated with different concentrations of PEA at 24 h and 72 h post-treatment compared to cells treated with Paclitaxel only, while at 48 h post-treatment only 0.1 µM PEA demonstrated a significant increase in neurite length against 1 µM Paclitaxel (* *p* < 0.05, ** *p* < 0.01, *** *p* < 0.001, **** *p* < 0.0001). Data are (mean ± SEM) of three independent experiments performed in 10–15 replicates. The asterisk denotes significant results regarding the respective measurement indicated with bar charts.

However, PEA concentrations (0.1, 1, and 10 µM) showed a significant protective effect on neurite outgrowth of DRG neurons when combined with 1 µM Paclitaxel and compared with 1 µM Paclitaxel alone at 24 and 72-h post-treatment. A total of 0.1 µM PEA combined with 1 µM Paclitaxel had a significant protective effect on neurite lengths of DRG neurons 48 h after treatment when compared to Paclitaxel alone (*p* < 0.05) (Figure 5).

**Figure 6:**
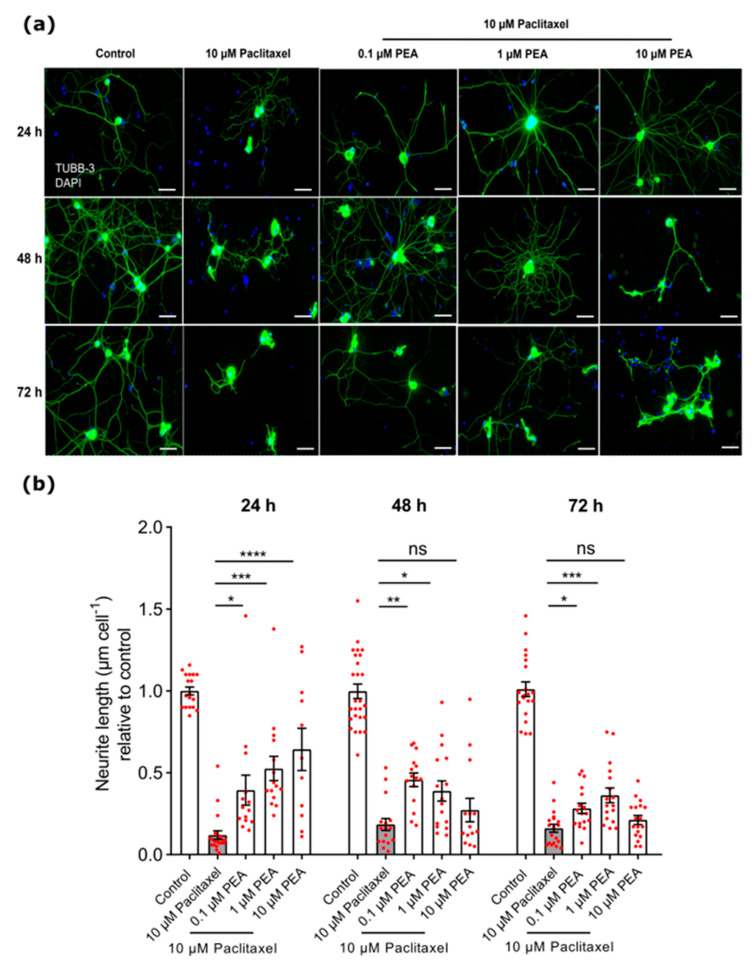
Effects of different PEA concentrations (0.1, 1, and 10 µM) combined with 10 µM Paclitaxel at 24, 48, and 72 h post-treatment. (**a**) Representative immunofluorescence images show DRG neurons labeled with beta III Tubulin antibody (green) and DAPI for nuclei (blue). Scale bars = 75 µm. (**b**) A significant increase in neurite length of neurons was found in groups treated with different concentrations of PEA at 24 h only compared to cells treated with Paclitaxel only. At 48 and 72-h post-treatment, 0.1 µM PEA or 1 µM PEA combined with 10 µM Paclitaxel demonstrated a significant increasing effect on the neurite lengths in comparison with 10 µM Paclitaxel alone (* *p* < 0.05, ** *p* < 0.01, *** *p* < 0.001, **** *p* < 0.0001). Data represented as (mean ± SEM), and the experiments were performed at least 3 independent times and 10–15 replicas. The asterisk denotes significant results regarding the respective measurement indicated with the bar graphs.

Regarding the protective effects of different PEA concentrations against 10 µM Paclitaxel, we found 0.1 or 1 µM PEA combined with 10 µM Paclitaxel showed a significant increase in neurite lengths of DRG neurons at 24, 48, and 72-h post-treatment compared to cells treated with 10 µM Paclitaxel only (*p* < 0.05) (Figure 6). Meanwhile, the 10 µM PEA plus 10 µM Paclitaxel group revealed a significant increase in neurite lengths only at 24 h post-treatment in comparison to the 10 µM Paclitaxel group (*p* < 0.05) (Figure 6).

#### 3.3.2. Soma Size

**Figure 7:**
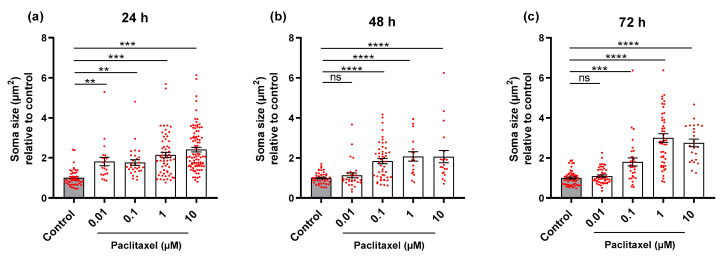
Effects of different Paclitaxel concentrations on soma size of DRG neurons at different time points. Bar charts show a significant increase in the soma size of DRG neurons after the application of different Paclitaxel concentrations at (**a**) 24 h, (**b**) 48 h, and (**c**) 72 h post-treatment compared to the control (** *p* < 0.01, *** *p* < 0.001, **** *p* < 0.0001). Asterisks denote significant results regarding the respective measurement indicated with the bar. Values are served as mean ± SEM of three independent experiments, n = 30–45 replicates. SEM: Standard error mean.

The four different concentrations of Paclitaxel led to an increase in the soma size of neurons 24 h post-treatment when compared to the control group (*p* < 0.05) (Figure 7a). At 48 and 72-h, all investigated groups treated with Paclitaxel showed apparent enlargements in areas of neuronal somata except for 0.01 µM of Paclitaxel when compared to the control group (*p* < 0.05) (Figure 7b,c). Effects of Paclitaxel on soma size of neuronal cell bodies were obviously time- and concentration-dependent. Treatment with PEA alone demonstrated no significant effects on the size of neuronal bodies at any time point when compared to control group (*p* > 0.05) (Figure S3d–f).

The effects of different combined groups of Paclitaxel plus PEA on soma size of DRG neurons were investigated in comparison to the cells treated with Paclitaxel alone. An increase in soma size was found for 0.01 µM Paclitaxel only at 24 h after treatment compared to control group. A total of 10 µM PEA combined with 0.01 µM Paclitaxel was the only group that demonstrated a significant decrease in neurons soma size in comparison to Paclitaxel group (*p* < 0.05) at 24 h after application, while 0.1 and 1 µM PEA did not show any protective effects against 0.01 µM Paclitaxel group (*p* > 0.05) at the same time point (Figure S6).

**Figure 8:**
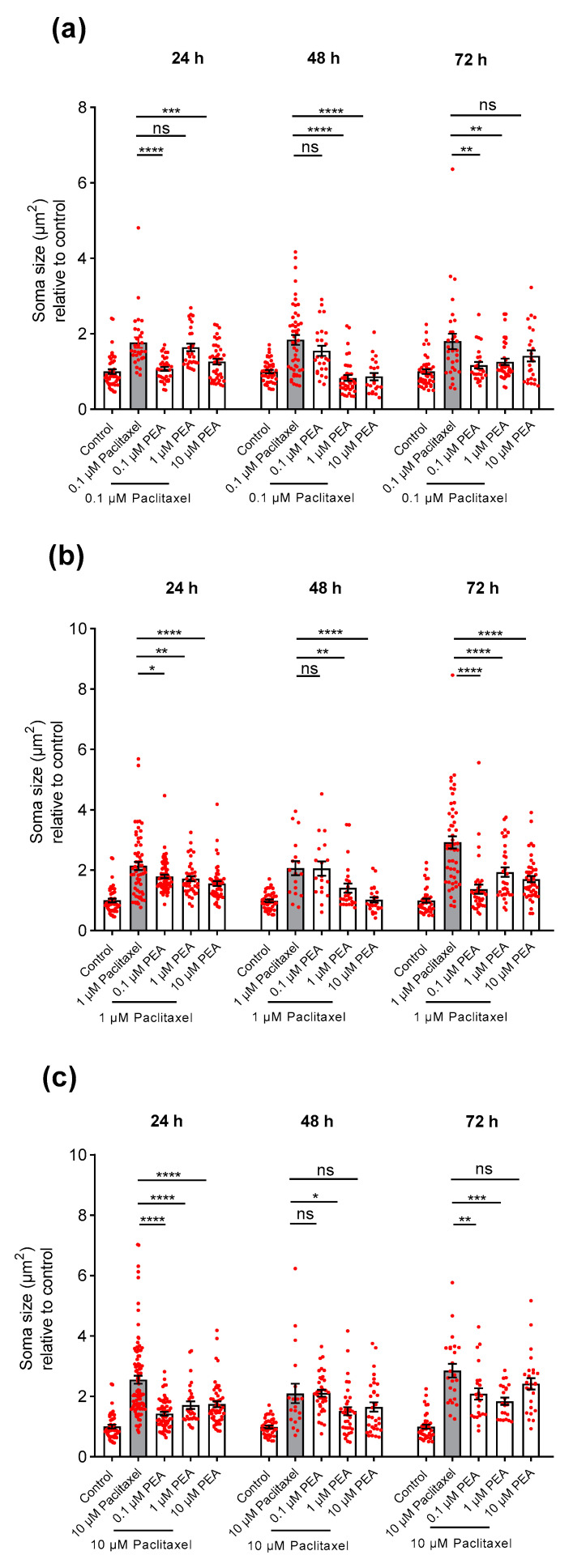
Effects of different PEA concentrations (0.1, 1, and 10 µM) in combination with different concentrations of Paclitaxel (**a**) 0. 1 µM, (**b**) 1 µM, and (**c**) 10 µM on soma sizes of DRG neurons at 24, 48, and 72-h post-treatment. The combined groups of Paclitaxel plus PEA demonstrated a varied significant decrease in DRG neuronal cell bodies in comparison with neurons treated with Paclitaxel only (* *p* < 0.05, ** *p* < 0.01, *** *p* < 0.001, **** *p* < 0.0001). Data represented as (mean ± SEM), and the experiments were performed at least 3 independent times with n = 30–45 replicas. The asterisk denotes significant results regarding the respective measurement indicated with the bar graphs.

The 0.1 and 10 µM PEA co-applied with 0.1 µM Paclitaxel revealed an apparent decrease in neuronal somata sizes, whereas 1 µM PEA had no effect compared to neurons treated with Paclitaxel only at 24 h after treatment (*p* < 0.05) (Figure 8a). At 48 h post-treatment, 1 and 10 µM of PEA combined with 0.1 µM of Paclitaxel were the only groups with a significant neuroprotective effect on somata sizes when compared to 0.1 µM Paclitaxel (*p* < 0.05) (Figure 8a). At 72 h after application, all PEA concentrations combined with 0.1 µM Paclitaxel showed considerable protectant action except for the 10 µM PEA + 0.1 µM Paclitaxel group when compared to Paclitaxel only (Figure 8a).

Treatment of neurons with the three different concentrations of PEA co-applied with 1 µM Paclitaxel demonstrated a significant protective effect on somata sizes when compared to cells exposed to 1 µM Paclitaxel alone at 24- and 72-h time points (*p* < 0.05) (Figure 8b). At 48 h post-treatment, 1 µM of Paclitaxel in combination with 10 µM of PEA was the only group without any protective effects, whereas the other two combined groups led to a strong decrease when compared to individual Paclitaxel treated cells (*p* < 0.05) (Figure 8b).

The three concentrations of PEA co-applied with 10 µM Paclitaxel showed statistically significant protective effects on DRG neurons’ cell bodies against the 10 µM Paclitaxel group at 24 h after treatment (*p* < 0.05) (Figure 8c). Similarly, both concentrations, 0.1 and 1 µM of PEA, combined with 10 µM of Paclitaxel showed a significant neuroprotective effect on soma sizes, while the combination of 10 µM Paclitaxel and 10 µM PEA did not have any protective effect at 72 h post-treatment compared to Paclitaxel alone (*p* < 0.05) (Figure 8c). The 10 µM Paclitaxel plus 1 µM PEA group demonstrated a significant protective effect; however, 0.1 and 10 µM of PEA combined with 10 µM Paclitaxel groups did not reveal any protective effect on the size of cell bodies of neurons when compared to neurons treated only with 10 µM Paclitaxel at 48 h after treatment (*p* < 0.05) (Figure 8c).

Overall, the neuroprotective actions of PEA against the induced toxicity of Paclitaxel on soma size of DRG neurons were time and concentration independent (*p* < 0.05).

## 4. Discussion

Peripheral neuropathy (PN) is one of the most common side effects of Paclitaxel, affecting up to 97% of all gynecological and urological cancer patients [46,47]. Paclitaxel causes cell death in cancer cells by interfering with mitosis via microtubule stabilization; however, Paclitaxel also affects the peripheral nervous system, causing PN [48]. The primary symptoms are hand and foot numbness besides pain caused by Paclitaxel accumulation in the DRG. DRG neurons are highly susceptible to Paclitaxel accumulation presumably due to a more permeable blood nerve barrier [49]. In the current study, the toxicity of Paclitaxel on viability of neurons was apparent at only 72 h post-treatment in comparison to the control group, while, at all-time windows studied, different Paclitaxel concentrations resulted in a significant reduction in neurite length of DRG neurons. These findings were in line with previous research on Paclitaxel-induced peripheral neuropathy with axonal sensory neuropathy that was length-dependent [50]. A significant reduction in neurite length was reported when DRG neurons were exposed to 10 µM Paclitaxel for 24 h [17]. In addition, Paclitaxel’s toxic effects on neurons resulted in the enlargement of neuronal cell bodies obviously at 24, 48, and 72 h post-treatment. These findings are in agreement with previous findings, in which Paclitaxel treatment caused a significant increase in DRG neuron soma size after 24 h of treatment [15,16] and induced a significant enlargement of DRG nucleolus size [51].

Our data obviously demonstrated that Paclitaxel neurotoxicity on neurite outgrowth and soma size is time- and dose-dependent. Similar earlier studies reported on a dose- and infusion time-dependent-induced neurotoxicity that could be exacerbated by underlying conditions or co-application with other drugs [52,53]. The differences in toxic effects of Paclitaxel on the viability and morphology of neurons might possibly be due to higher susceptibility or vulnerability of neurites to toxins than neuronal somata [15,16]. As a result, the toxic effects of Paclitaxel were more rapid, with a significant reduction in neurite length of neurons after 24 h post-treatment. The data are in agreement with a previous work reporting on reduction of axon length after Paclitaxel treatment. Therefore, Paclitaxel seems to act directly on axons and causes axonal degeneration probably through local mechanisms [54]. Additionally, Paclitaxel disrupted intracellular microtubules and bindings with beta-tubulin inside cell soma, resulting in accumulation of non-functional beta-tubulin units of microtubules, vacuolization of mitochondria and cytoplasm in neuronal cell bodies, and cell enlargement [55]. Paclitaxel increased cell size, and, after 72 h of treatment, neurons may explode and die due to a non-apoptotic effect. Taken together, Paclitaxel targets the nerve fibers and causes local axonopathy in still viable neurons with increased soma sizes.

PEA is a bioactive lipid that is used as an anti-nociceptive agent in different animal models of neuropathic pain, including spinal cord injury [56] and diabetes-induced peripheral neuropathy [57]. In humans, PEA accumulates in painful tissues, as observed in the trapezius muscle of women suffering from chronic neck pain [58]. Moreover, PEA protected nerve tissue in neuropathic conditions [37] and prevented neurotoxicity and neurodegeneration [59,60]. Furthermore, PEA also alleviated painful diabetic neuropathy, chemotherapy neuropathy, idiopathic axonal neuropathy, nonspecific neuropathy, and sciatic and lumbosacral spine disease pain [61]. Here, we demonstrated that PEA partially counteracted the toxicity of Paclitaxel on DRG neurons. Regardless of PEA concentration, combining PEA with Paclitaxel significantly increased neuron cell viability compared to treatment with Paclitaxel at 72 h after treatment. These results were consistent with a previous study that showed a reduction of positive propidium iodide (PI) neuronal nuclei after the application of PEA to N-methyl D-aspartate (NMDA)-treated organotypic hippocampal slice cultures [36]. The positive effects on cell viability seem not to be confined to neurons. In astrocytes, different PEA concentrations increased the cell viability from 30 min to 18 h [62]. The data imply for Paclitaxel the need for a longer interaction with damaged cells. Paclitaxel’s slow action might provide a good opportunity for PEA to exert protective effects and reverse the toxic effects of Paclitaxel, resulting in increasing the viability of DRG neurons.

In the present study, PEA plus Paclitaxel groups showed a significant increase in neurite length and a strongly decreased soma size of DRG neurons at all studied time points when compared to individual Paclitaxel treatment groups. Interestingly, at 24 h after treatment, PEA produced a protective effect on neurite length and size of cell bodies of neurons against toxicity of Paclitaxel, independent of PEA concentration. This phenomenon might be attributed to the short period of exposure of DRG neurons to Paclitaxel and PEA treatment, allowing PEA to mask and alleviate the toxicity of Paclitaxel. In line with previous evidence in a rat model of Oxaliplatin-induced neurotoxicity, acute intraperitoneal administration of PEA (30 mg kg-1) substantially relieved pain 30 min after administration [63].

Notably, 10 µM PEA did not show any significant protective effect on neurite outgrowth at 48 h post-treatment, although it enhanced neurite extension at 72 h after treatment. The results might be interpreted as an attempt of damaged neurons to develop a survival pressure to resist death caused by Paclitaxel toxicity. They retract and aggregate short neurites. Therefore, neurite extension might become a secondary process at 48 h post-treatment and, as a result, PEA remains unable to express any protective effects. These data are agreed with previous studies on PEA effects on preserving myelin sheet thickness and axonal diameter and preventing myelin degeneration [37]. PEA reduced myelin loss caused by sciatic nerve injury, maintained neuron cell diameters, reduced nerve edema, and restored nerve function, all of which were associated with decreased hypersensitivity [64].

In summary, PEA induced strong neuroprotective actions against Paclitaxel toxicity in DRG neurons and improved their viability and morphology.

## 5. Conclusions

Our findings showed the ability of PEA to attenuate the toxicity of Paclitaxel on DRG neurons. The effects of Paclitaxel on neuronal viability alone were apparent at 72 h post-treatment only. Furthermore, treatment with Paclitaxel led to a strong reduction in neurite length and enlargement of neuronal cell bodies at all investigated time windows. PEA showed neuroprotective effects by partially reversing the toxic effects of Paclitaxel, including increasing cell viability, enhancing DRG neuron neurite outgrowth, and decreasing swelling of neuronal soma. These findings contribute to our understanding of Paclitaxel’s site and mode of action on the peripheral nervous system and highlight the critical need for novel peripheral neuropathy protective strategies. More research will be needed to elucidate the signaling pathways underlying PEA’s neuroprotective effects against Paclitaxel neurotoxicity. With these results, PEA might be a promising therapeutic option for cancer patients suffering from CIPN.

## Data Availability

The data presented in this study are available on request from the authors.

## Supplementary Materials

The following supporting information can be downloaded at: https://www.mdpi.com/article/10.3390/biom12121873/s1. Figure S1: Representative example for tracing neurites by ImageJ program; Figure S2: Effects of different PEA concentrations on neuronal cell viability (%) (mean ± SEM) at 72 h post-treatment; Figure S3: Effects of different concentrations of PEA on neurite lengths and soma sizes (mean ± SEM) of DRG neurons at 24, 48, and 72-h post-treatment; Figure S4: Effects of different PEA concentrations (0.1, 1, and 10 µM) combined with 0.01 µM Paclitaxel on neurite length of DRG neurons at 24, 48, and 72-h post-treatment; Figure S5: Effects of different PEA concentrations (0.1, 1, and 10 µM) combined with 0.1 µM Paclitaxel at 24, 48, and 72-h post-treatment; Figure S6: The effects of different PEA concentrations (0.1, 1, and 10 µM) co-applied with 0.01 µM Paclitaxel on soma sizes of DRG neurons at 24, 48, and 72-h post-treatment.
