# Supplementary material for: Palmitoylethanolamide Mitigates Paclitaxel Toxicity in Primary Dorsal Root Ganglion Neurons
Source: Biomolecules. 2022 Dec 14;12(12):1873. doi: 10.3390/biom12121873 (PMC9775584; doi:10.3390/biom12121873)
Supplement: Supplementary file 1 [file biomolecules-12-01873-s001.zip › biomolecules-2082780-Supplementary Materials - clean.pdf]

Supplementary information

Palmitoylethanolamide Mitigates Neurotoxicity of Paclitaxel in Primary Dorsal Root Ganglion

Amira Elfarnawany[1,2] and Faramarz Dehghani[1*]

[1]Department of Anatomy and Cell Biology, Medical Faculty, Martin Luther University Halle-Wittenberg, Halle (Saale), Germany.

[2] Zoology Department, Faculty of Science, Tanta University 31527, Tanta, Egypt.

*Corresponding author: faramarz.dehghani@medizin.uni-halle.de (FD)

a)

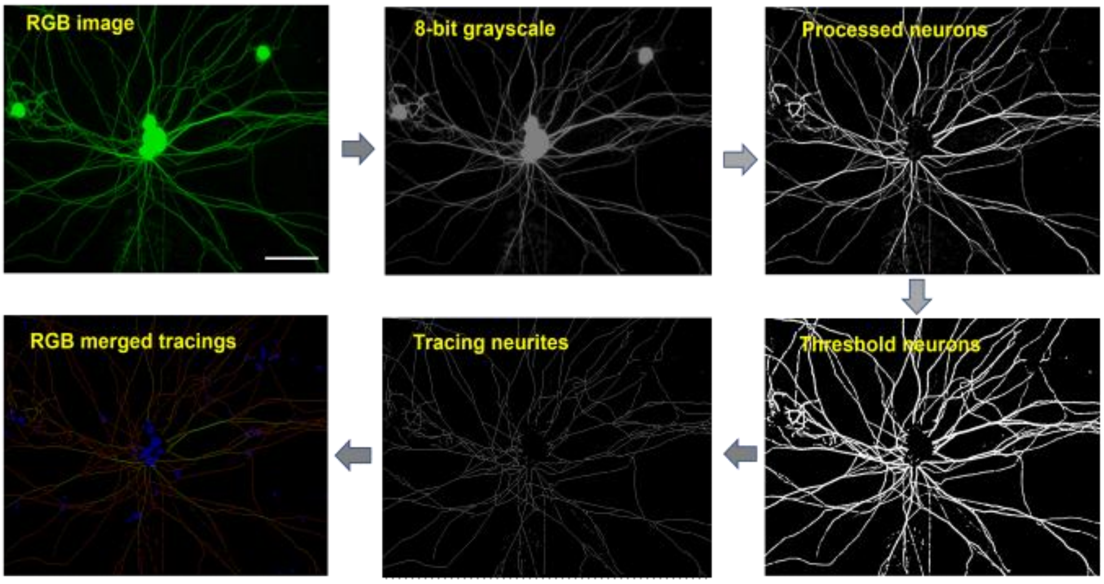

b)

A	B	C	D	E	F
	**Label**	**Length(pixels)**	**Length(µm)**	**Nu.cells**	**Length/cell**
**1**	**Neuronal-1.tif**	11703.555	44.27	1	44.27
**2**	**Neuronal-2.tif**	18245.877	69.01	1	69.01
**3**	**Neuronal-3.tif**	27843.286	105.32	2	52.66
**4**	**Neuronal-4.tif**	21344.411	80.73	2	40.365
	**average**	**24022.18**	**90.86**		**51.58**

Figure S1. Representative example for tracing neurites by ImageJ program for the control group **a)** Step-by-step Demonstration of neurite tracing and measurement of their length by automatic neurite tracer (ImageJ). Scale bars = 75 µm**. b**) Results of different neurite length measurements of sensory neurons for four different images for the same control group. Total neurite length in pixels converted to micrometers in the unit and normalized to the number of neurons per photo resulted in neurite length/cell.

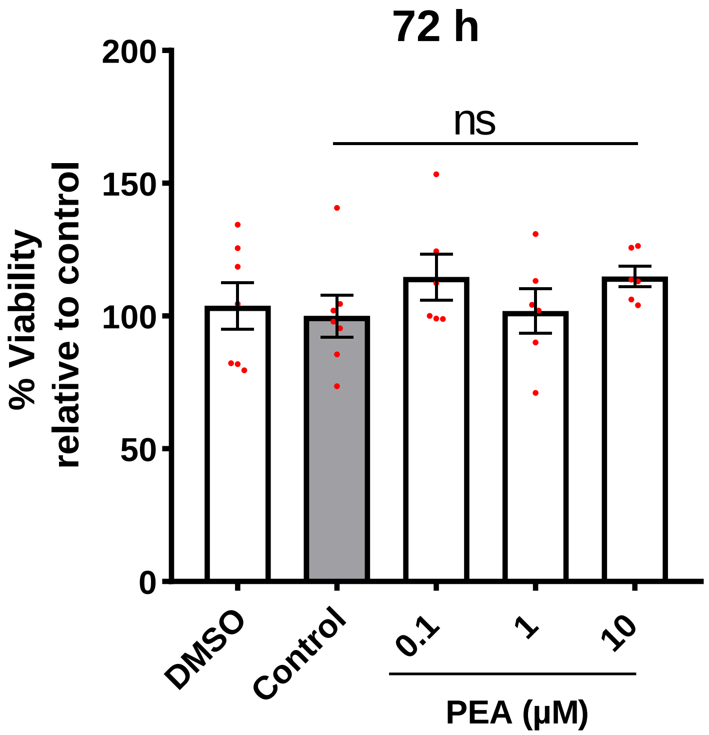

Figure S2. Effects of different PEA concentrations on neuronal cell viability (%) (mean ± SEM) at 72 h post-treatment. PEA showed no significant effect on the viability of cells compared to the control (p > 0.05). Experiments were performed 3 times independently 0f (6-9) technical replicates. SEM: standard error mean.

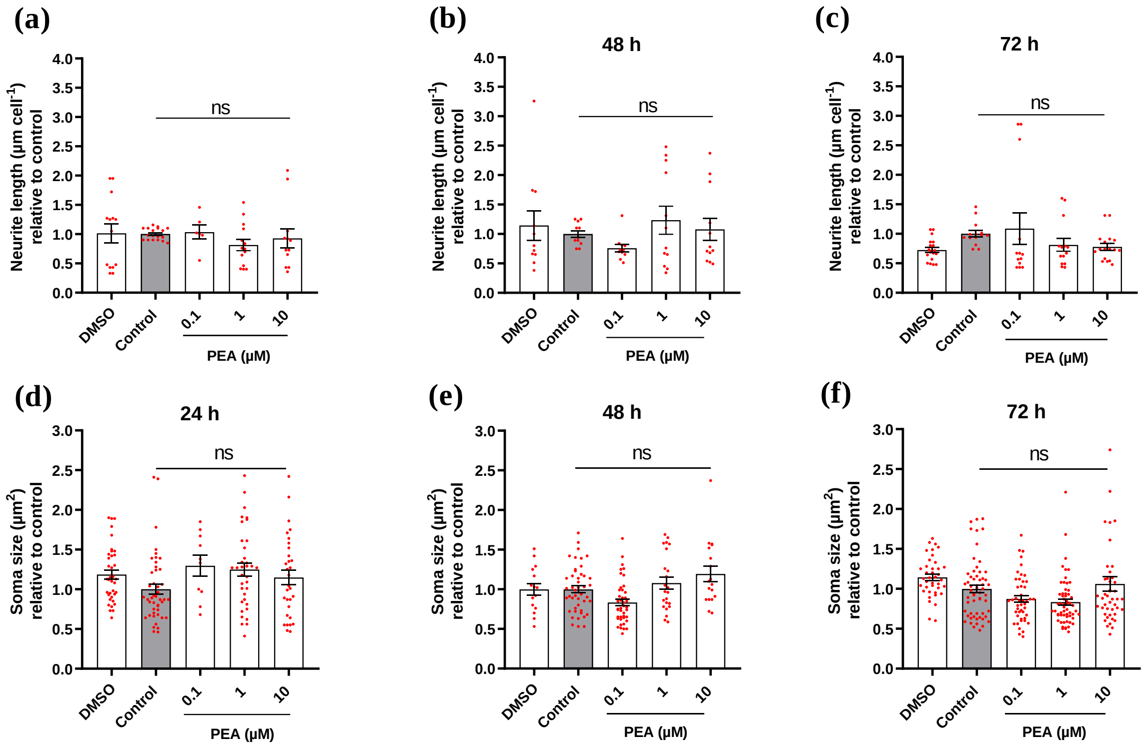

Figure S3. Effects of different concentrations of PEA on neurite lengths and soma sizes (mean ± SEM) of DRG neurons at 24, 48, and 72-h post-treatment. **a), b), and c)** No significant effect of PEA was found on the neurite lengths (n = 10-15 replicates) and **(d, e, and f)** soma sizes (n = 30-45 replicates) compared to control (p > 0.05). experiments were performed 3 times independently. SEM: standard error mean.

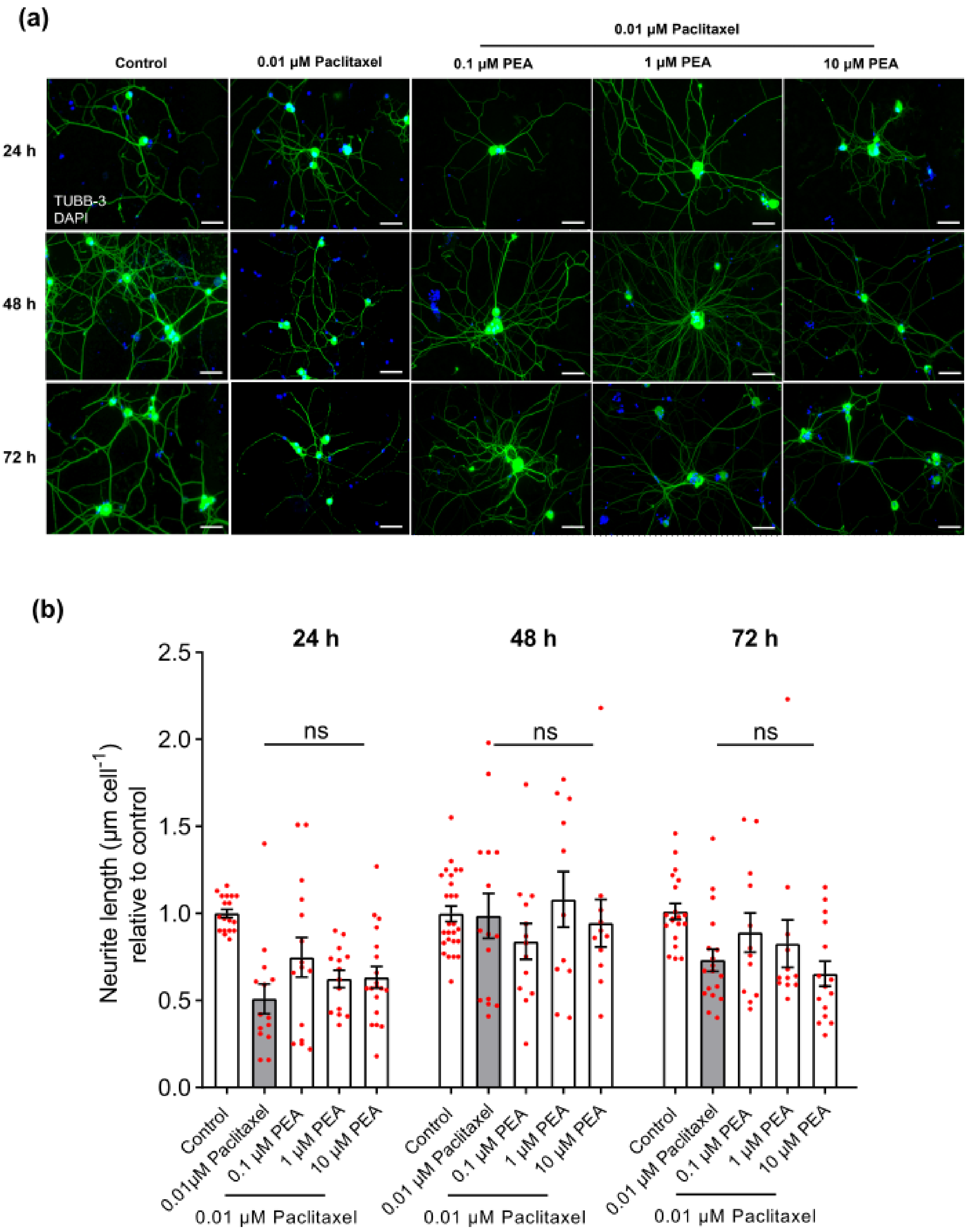

Figure S4. Effects of different PEA concentrations (0.1, 1, and 10 µM) combined with 0.01 µM Paclitaxel on neurite length of DRG neurons at 24, 48, and 72-h post-treatment **a)** representative images of DRG neurons labeled with beta III Tubulin antibody (green) and DAPI for nuclear staining (blue). Scale bars = 75 µm. **b)** No significant effects on neurite lengths were observed in DRG neurons treated with different concentrations of PEA at 24, 48, and 72h post-treatment compared to cells treated with only 0.01µM Paclitaxel (p > 0.05). Data represented (mean ± SEM) of N=3 independent experiments, n =10-15 replica.

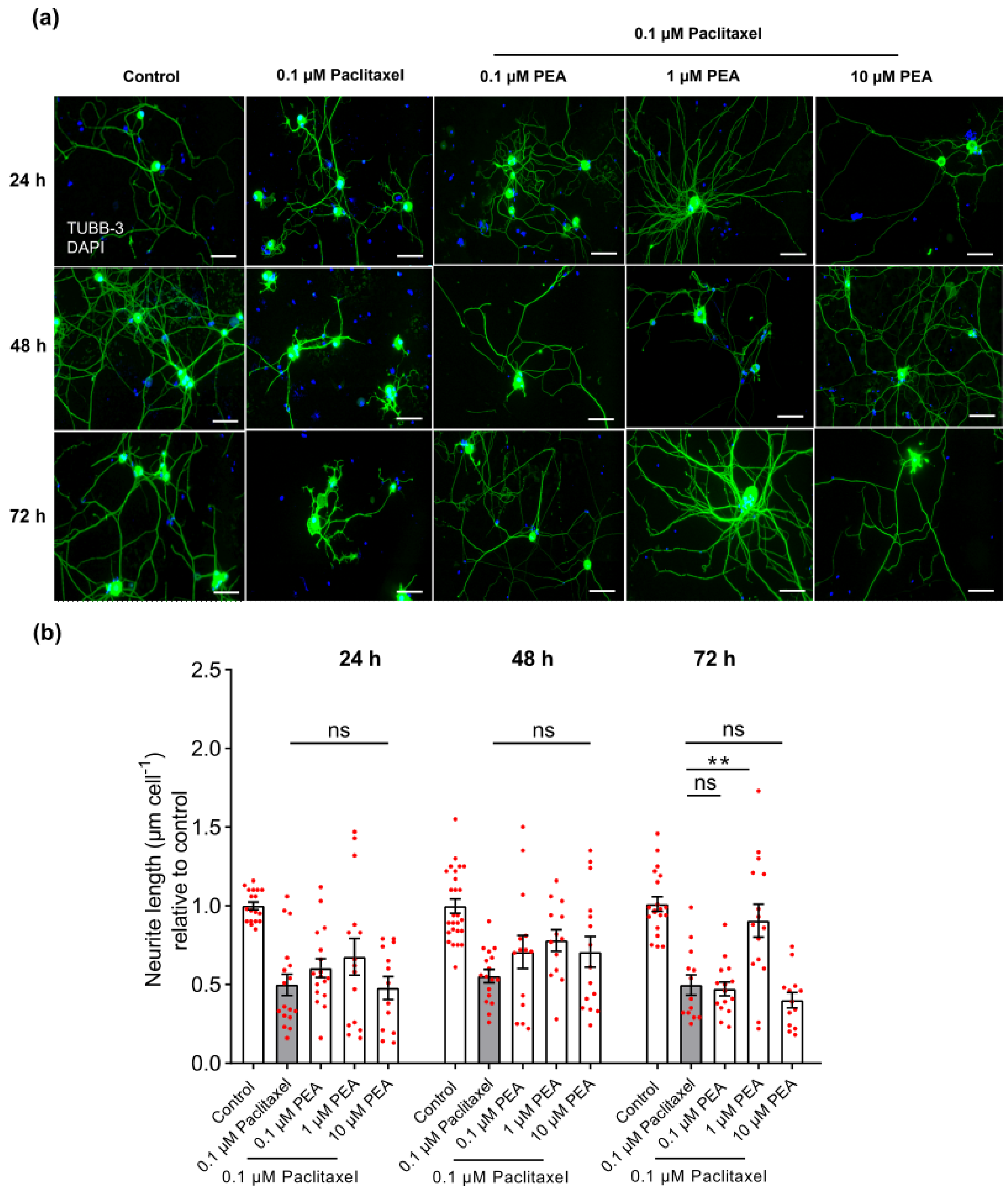

Figure S5. Effects of different PEA concentrations (0.1, 1, and 10 µM) combined with 0.1 µM Paclitaxel at 24, 48, and 72-h post-treatment **a)** representative images showed sensory neurons labeled with beta III Tubulin antibody (green) and DAPI for nuclear staining (blue). Scale bars = 75 µm. **b)** Bar graphs showed no significant effects on neurite lengths of DRGs neurons treated with different concentrations of PEA at 24, and 48-h of treatment compared to cells treated with only 0.01µM Paclitaxel (p > 0.05). 1 µM of PEA plus 0.1 µM Paclitaxel showed an apparent increase in neurite length of neurons against 0.1 µM Paclitaxel at only 72 h post-treatment (**p < 0.01). Data represented as (mean ± SEM), and the experiments were performed at least 3 independent times and 10-15 replicas. The asterisk denotes significant results regarding the respective measurement indicated with the bar graphs.

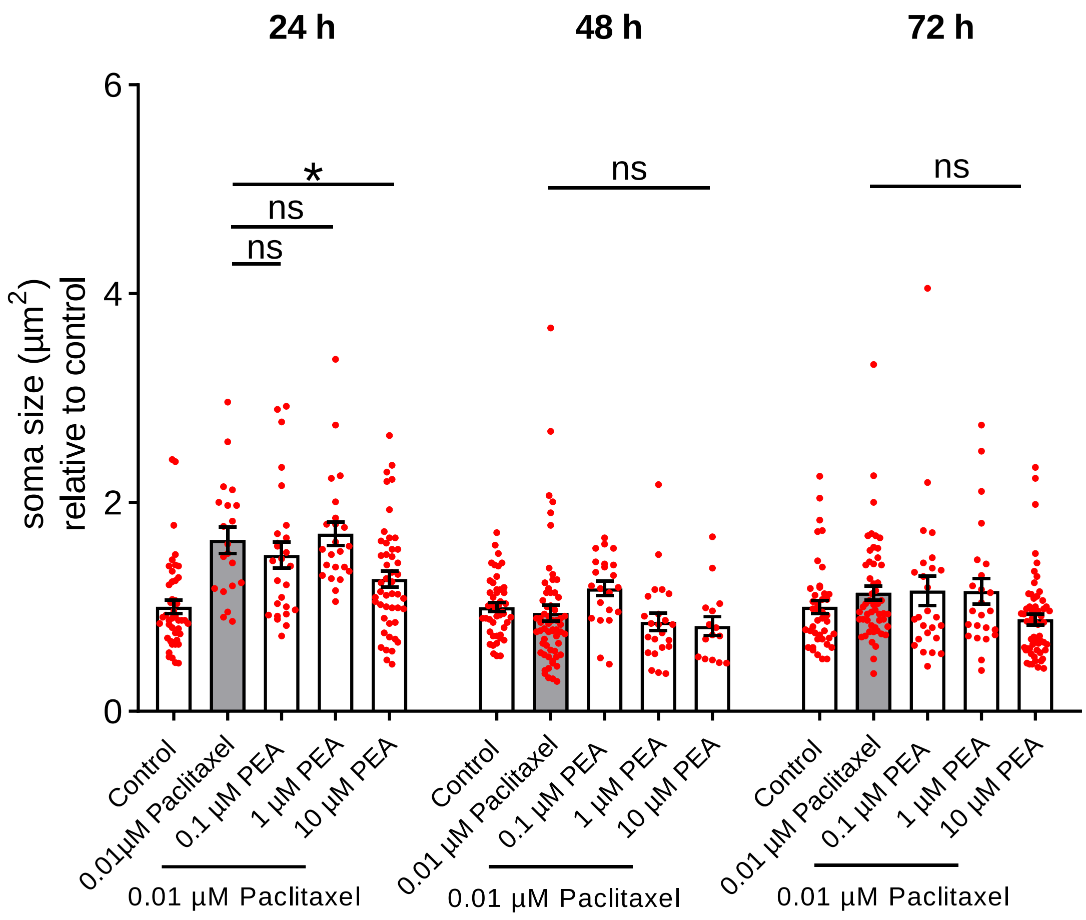

Figure S6. The effects of different PEA concentrations (0.1, 1, and 10 µM) co-applied with 0.01 µM Paclitaxel on soma sizes of DRG neurons at 24, 48, and 72-h post-treatment. At 24 h, only 10 µM PEA showed a significant decrease in soma size against 0.01 µM Paclitaxel alone (*p < 0.05), while there were no significant effects on soma sizes of DRG neurons treated with different concentrations of PEA at 48 and 72 h post-treatment compared to cells treated with 0.01µM Paclitaxel (p > 0.05). Data represented as (mean ± SEM), and the experiments were performed at least 3 independent times and 30-45 replicas. The asterisk denotes significant results regarding the respective measurement indicated with the bar graphs.
